# Enhanced lung inflammatory response in whole-body compared to nose-only cigarette smoke-exposed mice

**DOI:** 10.1186/s12931-021-01680-5

**Published:** 2021-03-17

**Authors:** Jef Serré, Ajime Tom Tanjeko, Carolien Mathyssen, An-Sofie Vanherwegen, Tobias Heigl, Rob Janssen, Eric Verbeken, Karen Maes, Bart Vanaudenaerde, Wim Janssens, Ghislaine Gayan-Ramirez

**Affiliations:** 1 Laboratory of Respiratory Diseases and Thoracic Surgery, Department of Chronic Diseases and Metabolism (CHROMETA), KU Leuven, Herestraat 49, O&NI bis, box 706, 3000 Leuven, Belgium; 2 Laboratory of Clinical and Experimental Endocrinology, Department of Chronic Diseases and Metabolism (CHROMETA), KU Leuven, Leuven, Belgium; 3 Department of Pulmonary Medicine, Canisius-Wilhelmina Hospital, Nijmegen, The Netherlands; 4 Translational Cell & Tissue Research, Department of Imaging & Pathology, KU Leuven, Leuven, Belgium

**Keywords:** COPD, Inflammation, Cigarette smoke, Nose-only, Whole-body

## Abstract

**Background:**

Chronic obstructive pulmonary disease (COPD) is characterized by a progressive and abnormal inflammatory response in the lungs, mainly caused by cigarette smoking. Animal models exposed to cigarette smoke (CS) are used to mimic human COPD but the use of different CS protocols makes it difficult to compare the immunological and structural consequences of using a nose-only or whole-body CS exposure system. We hypothesized that when using a standardized CS exposure protocol based on particle density and CO (carbon monoxide) levels, the whole-body CS exposure system would generate a more severe inflammatory response than the nose-only system, due to possible sensitization by uptake of CS-components through the skin or via grooming.

**Methods:**

In this study focusing on early COPD, mice were exposed twice daily 5 days a week to CS either with a nose-only or whole-body exposure system for 14 weeks to assess lung function, remodeling and inflammation.

**Results:**

At sacrifice, serum cotinine levels were significantly higher in the whole-body (5.3 (2.3–6.9) ng/ml) compared to the nose-only ((2.0 (1.8–2.5) ng/ml) exposure system and controls (1.0 (0.9–1.0) ng/ml). Both CS exposure systems induced a similar degree of lung function impairment, while inflammation was more severe in whole body exposure system. Slightly more bronchial epithelial damage, mucus and airspace enlargement were observed with the nose-only exposure system. More lymphocytes were present in the bronchoalveolar lavage (BAL) and lymph nodes of the whole-body exposure system while enhanced IgA and IgG production was found in BAL and to a lesser extent in serum with the nose-only exposure system.

**Conclusion:**

The current standardized CS-exposure protocol resulted in a higher internal load of serum cotinine in the whole-body exposure system, which was associated with more inflammation. However, both exposure systems resulted in a similar lung function impairment. Data also highlighted differences between the two models in terms of lung inflammation and remodelling, and potential sensitization to CS. Researchers should be aware of these differences when designing their future studies for an early intervention in COPD.

## Background

The prevalence and mortality of chronic obstructive pulmonary disease (COPD) is increasing and is now the third leading cause of mortality worldwide with approximately 65 million people suffering from moderate to severe COPD and 3 million deaths in 2016 [1]. COPD is characterized by a progressive and irreversible airflow limitation associated with an abnormal inflammatory response of the lungs to the inhalation of noxious particles and gases (e.g. tobacco smoke, pollution and greenhouse gases) [2]. Characteristics of COPD include an increased bronchial hypersecretion, narrowing and disappearance of the small airways and enlargement of airspaces resulting in a loss of elastic recoil. These features contribute to the chronic airflow limitation reflected by a progressive decline in forced expiratory volume in 1 s (FEV_1_) and a decrease in the Tiffeneau index < 70% (i.e. ratio of FEV_1_ to forced vital capacity (FVC)) [3]. Chronic airway inflammation is a key element in the pathophysiology of COPD as it contributes to parenchymal damage and destruction as well as to the narrowing of the small airways. COPD is characterized by a Type-1-mediated airway inflammation, with migration and activation of neutrophils, macrophages, helper T (Th) 1 and Th17 lymphocytes [4]. In particular, neutrophilic inflammation is highly related to tissue destruction and disease progression as their proteolytic enzymes cause alveolar elastin fiber breakdown with the loss of crosslinkers desmosine and iso-desmosine [5]. Chronic inflammation is normally expected to activate the draining lymph nodes, but surprisingly, enlarged hilar and mediastinal lymph nodes are only found in 49% of the COPD patients [6]. Persistent inflammation may also lead to the formation of lymphoid follicles in the lungs of COPD patients. Tertiary lymphoid follicles (TLF) are formed in response to chronic antigenic stimulation like persistent infections or chronic cigarette smoke (CS) inflammation. TLF are presumed to be involved in the acquisition and presentation of antigens locally and are associated with an increased production of local immunoglobulins in severe COPD-patients [7, 8]. They are strongly correlated with disease severity, but are not necessary present in mild to moderate COPD [9]. In summary, the mechanisms driving the abnormal chronic inflammation, initiation, structural alterations and impaired lung function are still not well understood.

As cigarette smoking is one of the major risk factors for developing COPD [2], several studies have examined the potential of animal models of chronic CS exposure to better understand the development of COPD. Up till now, CS exposure machines for animal models consist out of two delivery systems, namely via nose- and whole-body exposure [10]. In the former, animals are exposed to CS solely through their nose while in the latter, CS is delivered into a chamber in which the whole animal is exposed. In this case, the uptake of nicotine, tar and toxic substances from cigarette can probably also occurs through the skin, particularly when the animal is cleaning its fur. Although these systems represent an appropriate way to mimic COPD features, the absence of a standardized method and the diversity in the protocols used for animal exposure, make it difficult to compare the data obtained from these exposure systems. The protocols for CS exposure vary in terms of duration of exposure; amount of cigarettes/session, amount of sessions/day/week, cigarette brands (commercial or research), cigarettes filtered or without filter, type of smoke exposure (i.e. mainstream, side stream or a mixture of both) and way of exposure (i.e. intermittently by puffs like in humans or by a continuous flow) [10]. Moreover, the majority of these animal models refers to late COPD. A well-controlled standardized protocol of CS exposure using the nose-only and whole-body exposure systems within the same study is lacking, although this would allow comparing the key pathological elements of these delivery modes especially in the context of early development of COPD. Indeed, a model of early COPD is of great importance notably for drug interventions, as late stage COPD is associated with irreversible lung deterioration and difficulties to stop disease progression.

In the current study, we examined the structural and inflammatory responses of the lung in nose-only or whole-body CS exposed mice using a standardized protocol, designed to keep CS exposure conditions comparable between both systems. We intentionally chose for an early COPD mouse model while using 3 months of CS exposure. We hypothesized that mice exposed to CS with the whole-body system would experience a more severe inflammatory response, due to possible sensitization, uptake of gases through the skin or uptake of tar particles stuck at the skin via grooming. Subsequently, the more severe inflammatory response may lead to more lung remodeling and further impaired lung function.

## Materials and methods

### Animals

Male 8-week-old C57BL/6JolaH mice were purchased from Envigo (Horst, The Netherlands) and were housed in a conventional animal housing facility with a 12/12-h light–dark cycle. Animals were supplied with pelleted food and water ad libitum. All experiments were approved by the Ethical Committee of Animal Experiments of the KU Leuven (P147/2016).

### Cigarette smoke exposure

Mice were randomly divided into 3 groups: a nose-only CS exposed (n = 10), a whole-body CS exposed (n = 16) and an air exposed control group (n = 8). For the nose-only system, mice were placed in soft restraints and connected to a smoke exposure tower. The amount of mice in the nose-only CS exposure was calculated according to expected mortality according to previous studies while the amount of mice in the whole-body CS exposure was based on the expected higher risk for suffocation with this system. A computer-controlled puff was setup, exposing mice to 10 s CS followed by 50 s of room air. For whole-body exposure, each mouse was placed within its own compartment in the smoking chamber. Similarly, a computer-controlled puff generated 45 s of CS exposure and 15 s of room air. Smoking mice were exposed to mainstream CS from 3R4F research cigarettes with filter (Kentucky Tobacco Research and Development Center, University of Kentucky) using 6 cigarettes per session, two times a day, 5 days a week for 14 weeks (InExpose System, Scireq). Each cigarette yields a concentration of 9.4 mg tar, 0.73 mg nicotine and 12.0 mg CO. This protocol was chosen to obtain the same average particle density of 188 ± 28.4 mg/m^3^ (Microdust, Casella CEL, Bedford, UK) and CO levels (respectively 871 ± 49 vs 937 ± 50 ppm/cigarette) (Easylog CO meter, Lascar electronics, Wiltshire, UK) with both exposure systems. Control mice were exposed to room air for the same duration.

### Lung function

After 14 weeks, mice were anaesthetized intraperitoneally with a xylazine (8.5 mg/kg, Rompun, Bayer, Belgium) and ketamine (130 mg/kg, Ketalar, Pfizer, Belgium) mixture 12 h after the last exposure to CS or room air. Tracheotomized mice were placed in a whole-body plethysmograph (Buxco—Force Pulmonary Maneuvers) to determine Functional Residual Capacity (FRC) through the Boyle’s law maneuver, Inspiratory Capacity (IC), Forced Vital Capacity (FVC), Total Lung Capacity (TLC) with the quasi-static pressure volume maneuver (PV-loop), the Forced Expiratory Volume in 100 ms (FEV100) and chord compliance between 0 and 10 cmH_2_O (Cchord) with the fast volume maneuver (FV-loop), as previously described [11].

### Bronchoalveolar lavage

After lung function, bronchoalveolar lavage (BAL) was performed with 1 × 500 μl and 3 × 1000 μl saline (B. Braun Medical, Diegem, Belgium). The supernatant of the first fraction was collected separately and used to perform cytokine analysis, while cells were pooled together to determine total cell counts using a Bürker hemocytometer with trypan blue. For differential cell counts, BAL cells were centrifuged (300 g, 6 min) onto microscope slides (Cytospin, Shandon, TechGen, Zellik, Belgium) and stained with Diff-Quick® (Medical Diagnostics, Düdingen, Germany). For each slide, 3 times 100 cells were counted to determine the percentage of macrophages, neutrophils and lymphocytes.

### Lung histopathology

The heart-left lung block was fixated in 6% paraformaldehyde at a constant hydrostatic pressure of 25 cm fluid column for 24 h. The left lung was collected, dehydrated and embedded in paraffin. Sagittal sections were stained with H&E and scored for inflammation and morphological changes in a blinded fashion for each functional compartment separately e.g. broncho-vascular bundle, alveolar parenchyma and venous compartment (E.V.). The broncho-vascular bundle was scored for epithelial denudation, diffuse inflammation infiltrates, vascular lymphoid aggregates and mucus in the airways. The alveolar compartment was scored for pigmented macrophages, lymphoid aggregates and diffuse inflammation. The venous compartment was scored for the presence of lymphoid aggregates and diffuse inflammation. Lymphoid aggregates and diffuse inflammation were averaged per lung slide. Scoring ranged from 0 to 3: 0 = normal, 0.5 = minimal, 1 = mild, 2 = moderate and 3 = severe. Airspace enlargement was quantified in 10–15 fields per lung, taken structurally randomized, at a magnification of × 200 by measuring the mean linear intercept (Lm) using an in-house macro in ImageJ.

### Serum analysis

Blood was collected from the vena cava and kept at room temperature for 1 h. Serum was collected and stored at − 20 °C. Cotinine levels were measured with a competitive ELISA according to manufacture instructions (KA2264, Abnova, Taipei City, Taiwan). Desmosine and iso-desmosine levels were measured with a liquid chromatography-mass tandem-mass spectrometry (LC–MS/MS), as previously described [12, 13]. Immunoglobulin (Ig) A and IgG were measured with a sandwich ELISA according to instructions (Ready-Set-Go!®, Invitrogen, Merelbeke, Belgium).

### Flow cytometric analysis of lymph nodes

Cervical and mediastinal lymph nodes were collected separately and kept on ice in medium (RPMI-1640 (1x) + GlutaMAX-1) (Invitrogen, Merelbeke, Belgium). A single cell suspension was obtained by pressing the lymph nodes through a cell strainer of 100 µm (BD Bioscience, Erembodegem, Belgium) and rinsing the cell strainer with medium. Cells were centrifuged (1000 g, 10 min at 4 °C) and suspended in 1 ml medium and counted using a Bürker hemocytometer. Cells were centrifuged (1000 g, 10 min at 4 °C) again and suspended in medium at a concentration of 10^7^ cells/ml. 500,000 cells were stained for 30 min with either a combination of anti-CD3 (APC)-, anti-CD4 (APC-Cy7)- and anti-CD8 (PerCP-CY5.5)- or only with anti-CD19 (PE)-labeled antibodies (BD Biosciences, Erembodegem, Belgium). Cells were washed with 1 × PBS and suspended in MACS buffer (1 × PBS, 0.5% BSA, 2 mM EDTA). Percentages of labeled cells were assessed by flow cytometry (KantoII, BD Biosciences, Erembodegem, Belgium) and analyzed in FlowJo (FlowJo LLC, Ashland, Oregon) on at least 10^5^ cells.

### Expression of inflammatory mediators and proteases/anti-proteases

The right lung was snap-frozen in liquid nitrogen and stored at − 80 °C. Total RNA was extracted by homogenizing the lung in TRIzol® (ThermoFisher scientific, Life Technologies, Waltham, MA USA) and using the RNAeasy mini kit (Qiagen, Leudsen, Netherlands) according to manufacturer’s instructions. 1 µg of RNA was reverse transcribed with Superscript III reverse transcriptase (Invitrogen, Life Technologies, Lennik, Belgium) in a total reaction of 20 µl. Random hexametric primers were used for cDNA synthesis at 42 °C for 50 min, followed by 15 min incubation at 70 °C. The quantitative PCR amplification reaction was performed in a total volume of 10 µl using Platinum®SYBR® Green qPCR SuperMix-UDG with a thermal cycler (Eco Real-Time PCR system, Illumina). Ribosomal protein L27 (RPL27) was used as housekeeping gene and data were normalized using the comparative cycle threshold (∆∆CT) method. All primers were in the 90–105% primer efficiency range. We examined the expression of main pro-inflammatory cytokines and the misbalance between proteases and anti-proteases. Primers are shown in Table 1. In addition, we measured IL-6, CXCL1, MCP-1 and TNF-α proteins with an MSD U-plex Elisa (Meso Scale Discovery®, Rockville, USA) in the first BAL fraction and serum.

**Table 1:** Primer sequences

Target	Forward primer	Reverse primer	Accession ID
RPL27	5′-GTCGAGATGGGCAAGTTCAT-3′	5′-TTCTTCACGATGACGGCTTT-3′	NM_011289.3
MMP12	5′-TTTTGATGGCAAAGGTGGTA-3′	5′-GCCTCATCAAAATGTGCATC-3′	NM_001320077.1 NM_001320076.1 NM_008605.3
TIMP1	5′-GTGGGAAATGCCGCAGAT-3′	5′-GGGCATATCCACAGAGGCTTT-3′	NM_001044384.1 NM_011593.2 NM_001294280.2
CXCL1	5′-ACCGAAGTCATAGCCACACTC-3′	5′-TCTCCGTTACTTGGGGACAC-3′	NM_008176.3
TNF-α	5′-CTGAGTTCTGCAAAGGGAGAG-3′	5′-CCTCAGGGAAGAATCTGGAAAG-3′	NM_013693.3 NM_001278601.1
IFN-γ	5′-AGCGGCTGACTGAACTCAGATTGTAG-3′	5′-GTCACAGTTTTCAGCTGTATAGGG-3′	NM_008337.4
IL-6	5′-TAGTCCTTCCTACCCCAATTTCC-3′	5′-TAGTCCTTCCTACCCCAATTTCC-3′	NM_031168.2
IL-12p40	5′-GGACCAAAGGGACTATGAGAAG-3′	5′-CTTCCAACGCCAGTTCAATG-3′	NM_001303244.1
IL-17	5′-GCTGGACCACCACATGAA-3′	5′-GCATCTTCTCGACCCTGAAA-3′	NM_010552.3
IL-23	5′-GGCATCGAGAAACTGTGAGAA-3′	5′-AGCCACCCAGGAAAGTATAAT-3′	NM_031252.2
IL-4	5′-CCCCAGCTAGTTGTCATCCTG-3′	5′-CAAGTGATTTTTGTCGCATCCG-3′	NM_021283.2
IL-13	5′-CAGCCTCCCCGATACCAAAAT-3′	5′-GCGAAACAGTTGCTTTGTGTAG-3′	NM_008355.3
IL-10	5′-GCTCCAAGACCAAGGTGTCTACAA-3′	5′-CCGTTAGCTAAGATCCCTGGATCA-3′	NM_010548.2
TGF-β	5′-CTGAACCAAGGAGACGGAATAC-3′	5′-GGGCTGATCCCGTTGATTT-3′	NM_011577.2

*RPL27* ribosomal protein L27, *MMP12* matrix metalloproteinases 12, *TIMP1* tissue inhibitor of matrix metalloproteinase, *CXCL1* C-X-C motif ligand (CXCL) 1, *TNF-α* tumor necrosis factor-α, *IFN-γ* interferon gamma, *IL* interleukin, *TGF-β* tumor growth factor beta

### Statistical analysis

Datasets were analyzed using GraphPad Prism 7.04 for windows (GraphPad Software, San Diego, USA) and are presented as median and IQR. Data was analyzed with a one-way ANOVA with Bonferroni post-hoc test or Kruskal–Wallis with Dunn’s test for multiple comparison depending on respectively parametric or non-parametric datasets. Differences were considered significant when p-values were less than 0.05.

## Results

### Cotinine serum levels

Serum levels of Cotinine were significantly increased in the whole-body exposure system (5.3 (2.3–6.9) ng/ml) compared to control (1.0 (0.9–1.0) ng/ml, p = 0.0004) and nose-only (2.0 (1.8–2.5) ng/ml, p = 0.004). The slight increase in serum cotinine levels in nose-only exposure system compared to controls failed to reach statistical significance.

### Lung function

**Fig. 1:**
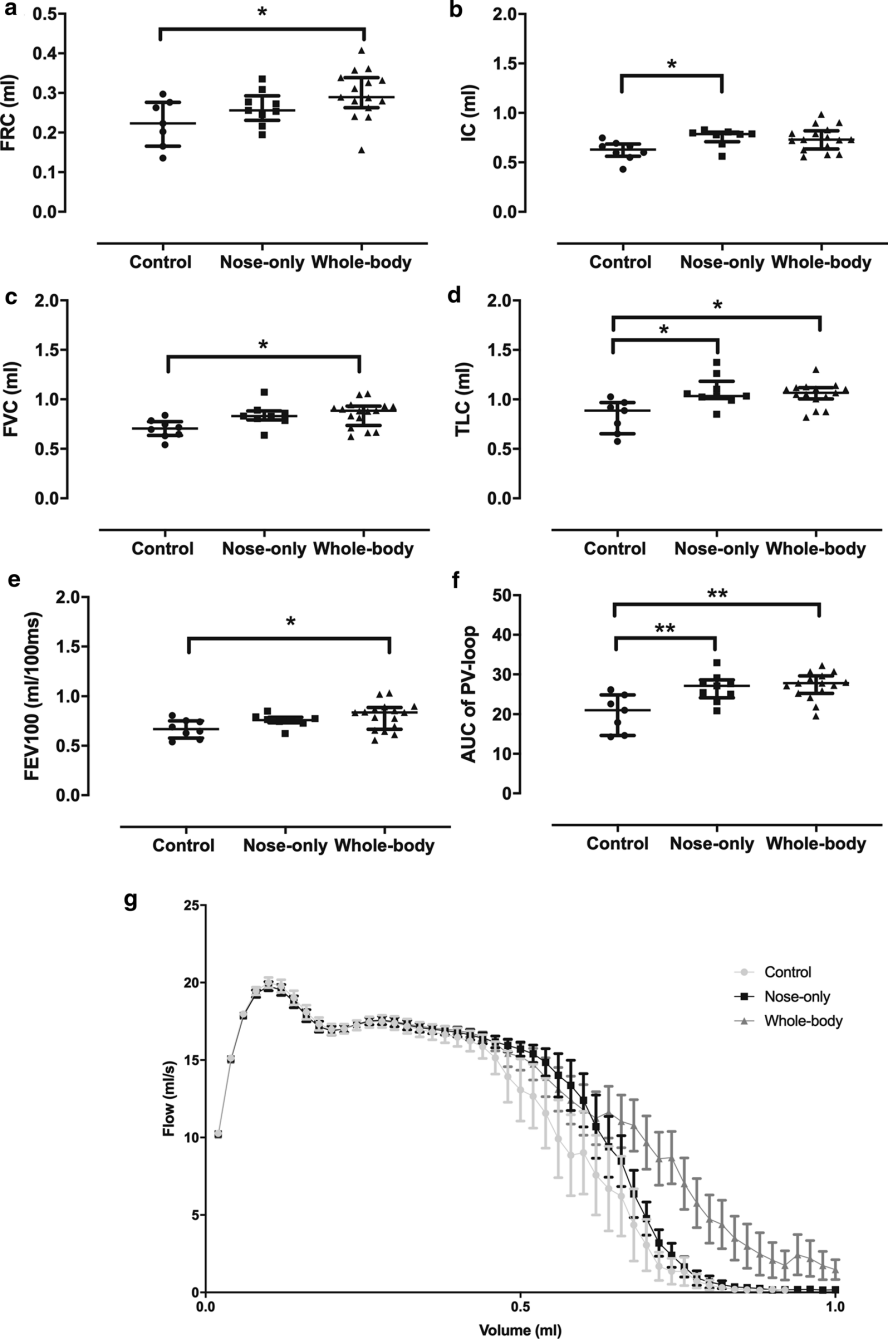
Lung function measurement of mice exposed to either air or CS in a nose-only or whole-body system. **a** Functional residual capacity (FRC), **b** inspiratory capacity (IC), **c** forced vital capacity (FVC), **d** Total lung capacity (TLC), **e** Forced expiratory volume in 100 ms (FEV100), **f** area under the curve (AUC) of the Pressure–Volume (PV)-loop (**G**) and the Flow-Volume (FV) loop was measured. *p < 0.05; **p < 0.01. Data are expressed as median and IQR (n = 8–15)

Whole-body CS exposed mice showed a significant increase in FRC (34%, p = 0.031) compared to controls, but not in the nose-only (17%) exposure system. Furthermore, there were no statistical differences between the two CS exposure systems (Fig. 1a). Compared to controls, IC was enhanced to the same extent (20%) in both CS-exposed groups (nose-only: p = 0.043, whole-body: p = 0.068) (Fig. 1b) as was FVC (nose-only: 20%, p = 0.084 and whole-body: 22%, p = 0.017) (Fig. 1c). In addition, TLC was significantly and similarly increased with both CS exposure systems (nose-only: 31%, p = 0.034 and whole-body: 27%, p = 0.012) (Fig. 1d) as was the chord compliance (20%, data not shown). The FEV100 was significantly higher with the whole-body exposure system (19%, p = 0.04), whereas nose-only mice showed a slight enhancement (12%) compared to controls (Fig. 1e). Whole-body exposed mice demonstrated a longer expiratory flow on the FV-loop in comparison with other groups (Fig. 1g), although the area under the curve (AUC) was similar between the different groups (data not shown). The PV-loop showed a clear upwards and left shift with a significant increase in the AUC in both CS exposure systems compared to control mice (nose-only: 32%, p = 0.0066 and whole-body: 34%, p = 0.0012; Fig. 1f).

### Structural changes in the lung

**Fig. 2:**
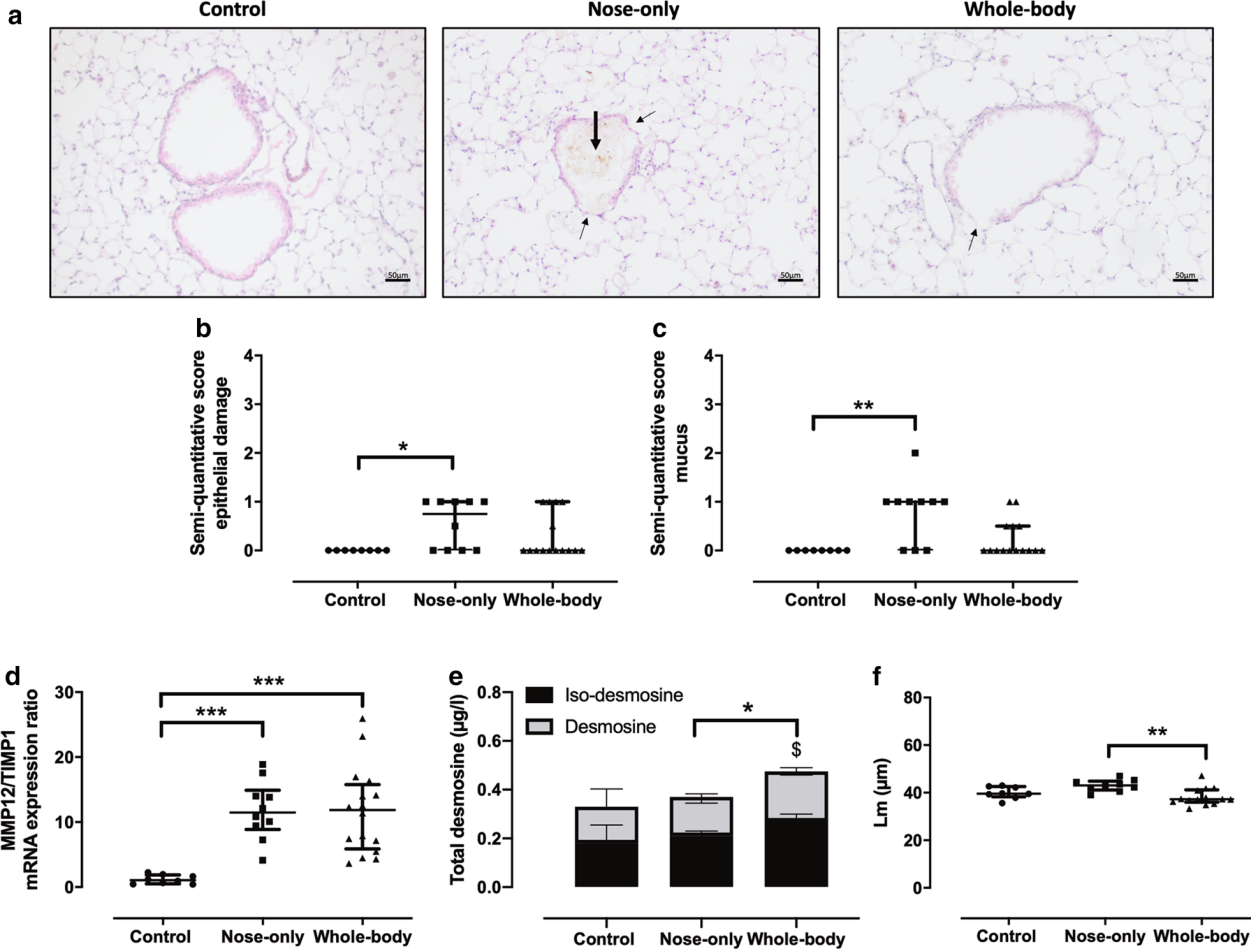
Lung remodeling in air and CS-exposed mice. **a** H&E staining of lung sections (× 200) from a representative control and CS-exposed mouse of the nose-only and whole-body system. Semi-quantitative score of **b** epithelial damage (thin arrow) and **c** mucus production (thick arrow). Pre- and post-elastin degradation markers were determined via **d** the MMP12/TIMP1 mRNA expression ratio and **e** total serum desmosine (*p < 0.05; nose-only vs whole-body) and iso-desmosine (^$^p < 0.05; control vs whole-body). **f** Airspace enlargement (Linear mean intercept, Lm) was measured with an in-house program. *p < 0.05; **p < 0.01; ***p < 0.001. Data are expressed as median and IQR (n = 8–15)

Bronchial epithelial damage (Fig. 2a, thin arrows) and mucus production (Fig. 2a, thick arrows) were observed in the lungs of CS exposed groups, but this was significantly more pronounced in the nose-only CS exposure system compared to controls, as shown on the semi-quantitative scoring (respectively p = 0.0261 and p = 0.0035, Fig. 2b, c). The MMP12/TIMP1 mRNA expression ratio in the lung was significantly elevated to the same extent in both CS exposure systems compared to controls (Fig. 2d). The total desmosine (i.e. desmosine and iso-desmosine) fraction in the serum was significantly enhanced in whole-body compared to the nose-only system (26%, p = 0.012), the latter showing similar values as to control (Fig. 2e). The iso-desmosine serum levels in the whole-body CS exposure system were significantly increased compared to control (p = 0.026), whereas the desmosine serum levels were significantly higher compared to nose-only (p = 0.031). In BAL, iso-desmosine and desmosine levels were under the detection limit. Airspace enlargement (Lm) was significantly enhanced with the nose-only CS exposure system, but solely in comparison with the whole-body CS exposure system (Fig. 2f).

### Histological evaluation of lung inflammation

**Fig. 3:**
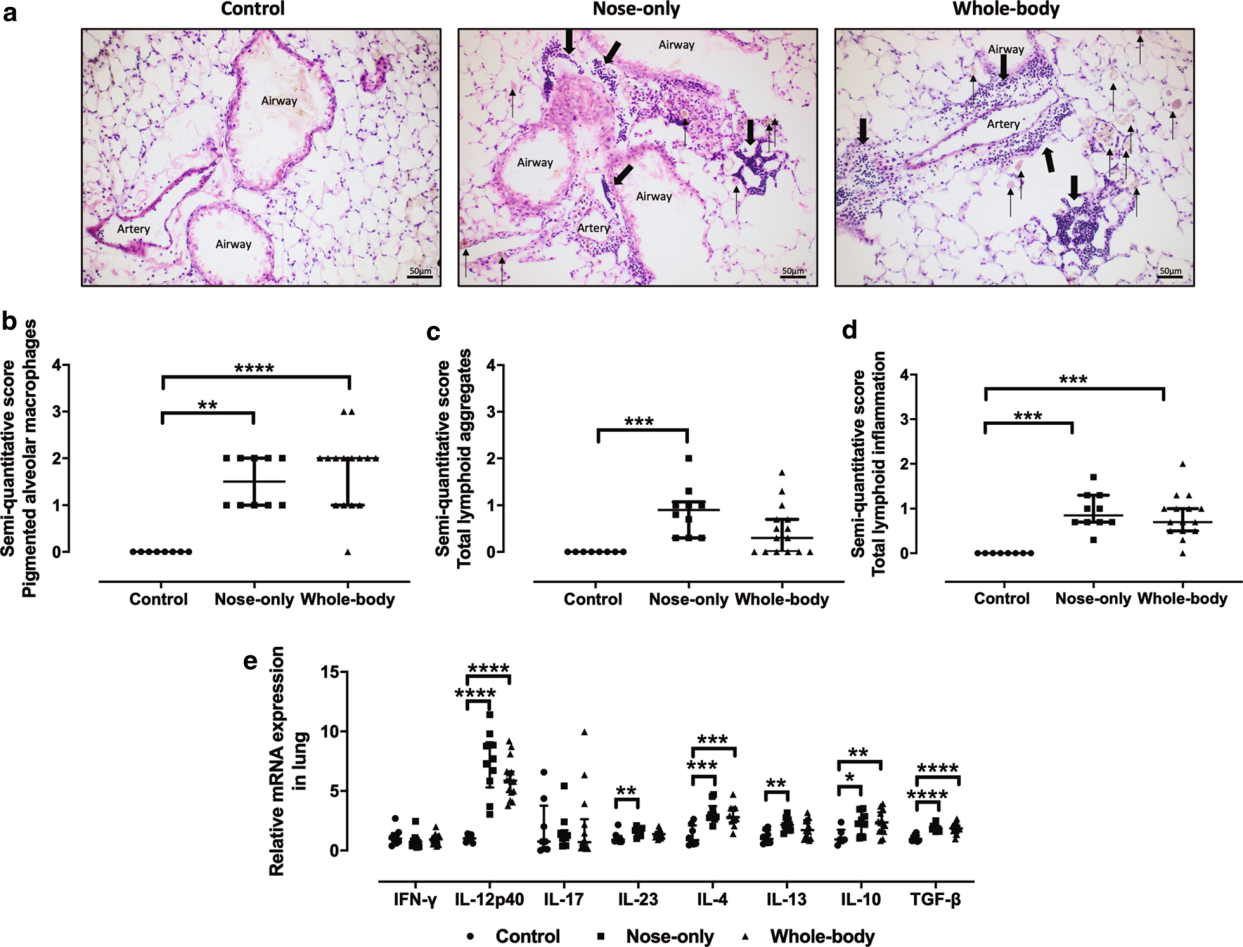
Histological evaluation of lung inflammation. **a** H&E staining of lung slides (× 200) of a representative nose-only and whole-body mouse lung slide. Semi-quantitative scoring of **b** pigmented alveolar macrophages (thin arrows), **c** lymphoid aggregates (thick arrows) and **d** diffuse lymphoid inflammation. **e** Relative mRNA expression of interferon (IFN)-γ, interleukin (IL)-12p40, IL-17, IL-23, IL-4, IL-13, IL-10 and transforming growth factor (TGF)-β. *p < 0.05; **p < 0.01; ***p < 0.001; ****p < 0.0001. Data are expressed as median and IQR (n = 8–15)

Compared to controls, a significantly higher quantity of pigmented alveolar macrophages was found in both CS exposure systems (Fig. 3a , b, thin arrows). In addition, lymphoid aggregates were present around veins and broncho-vascular bundles (Fig. 3a, thick arrows), however, this was only statistically significant with the nose-only CS exposure system (Fig. 3c). Lymphoid inflammation was similarly and significantly enhanced with both systems in comparison to control (Fig. 3d). Both CS exposure systems induced an enhanced cytokine mRNA expression in lung homogenate, with an increased expression of IL-12p40, IL-4, IL-10 and TGF-β compared to control (Fig. 3e). Only the nose-only exposure system induced a significant higher expression of IL-23 and IL-13 in comparison with control, hence almost the same increase was found in the whole-body system (Fig. 3e). IFN-γ and IL-17 mRNA expression remained unchanged (Fig. 3e).

### Airway inflammation in BAL

**Fig. 4:**
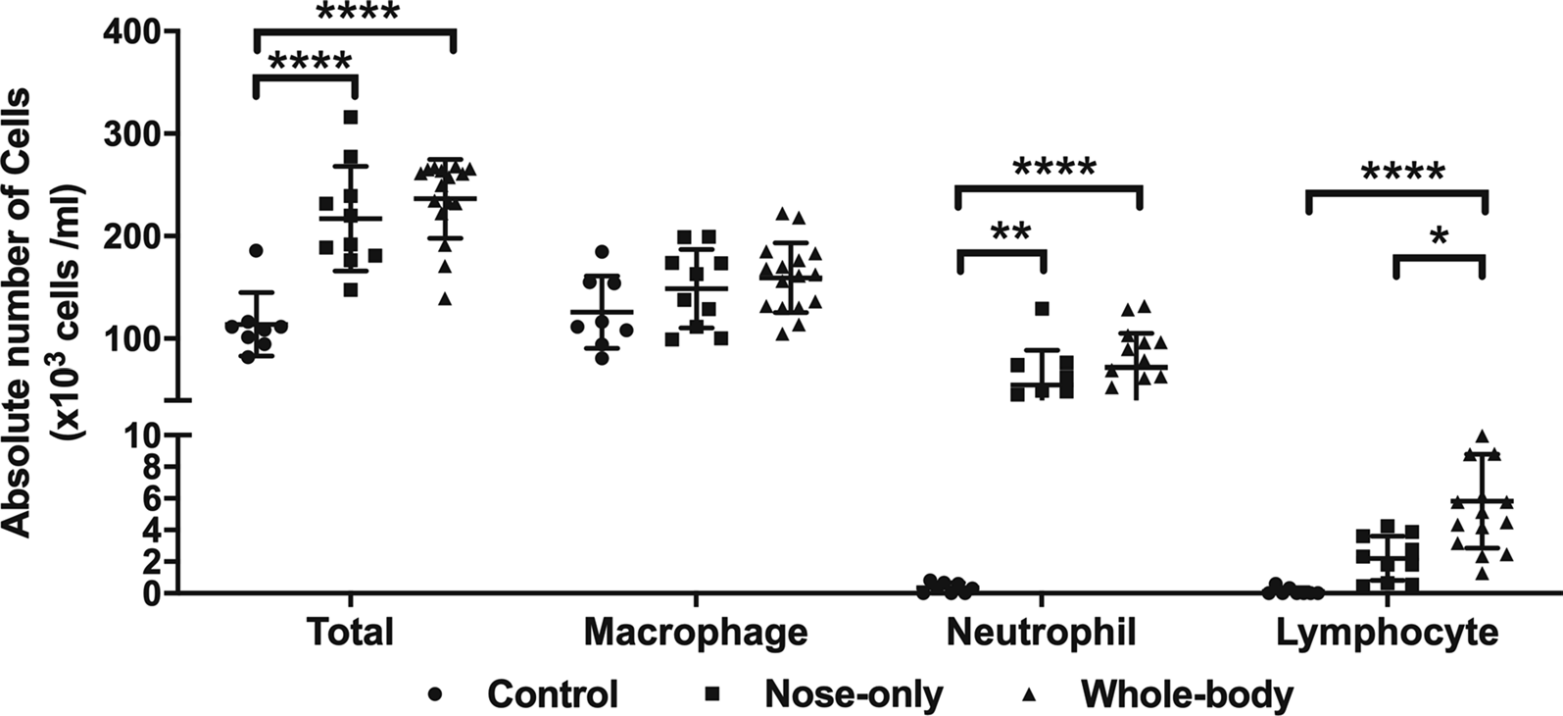
Airway inflammation measured in BAL. Absolute number of total cells, macrophages, neutrophils and lymphocytes. *p < 0.05; **p < 0.01; ****p < 0.0001. Data are expressed as median and IQR (n = 8–15)

The absolute number of leukocytes in BAL was enhanced by twofold in both CS exposure systems compared to controls. This enhanced leukocyte influx was predominately driven by neutrophils which were increased by 24% (p = 0.007) and 30% (p < 0.0001) respectively in the nose-only and whole-body system, while the number of macrophages remained unchanged (Fig. 4a). In contrast, the absolute number of lymphocytes in BAL was only significantly elevated in the whole-body CS exposure system compared to controls (2.4%, p < 0.0001) and nose-only (1.3%, p = 0.0346) (Fig. 4).

### Pro-inflammatory cytokines and chemokines in BAL, serum and lung

**Fig. 5:**
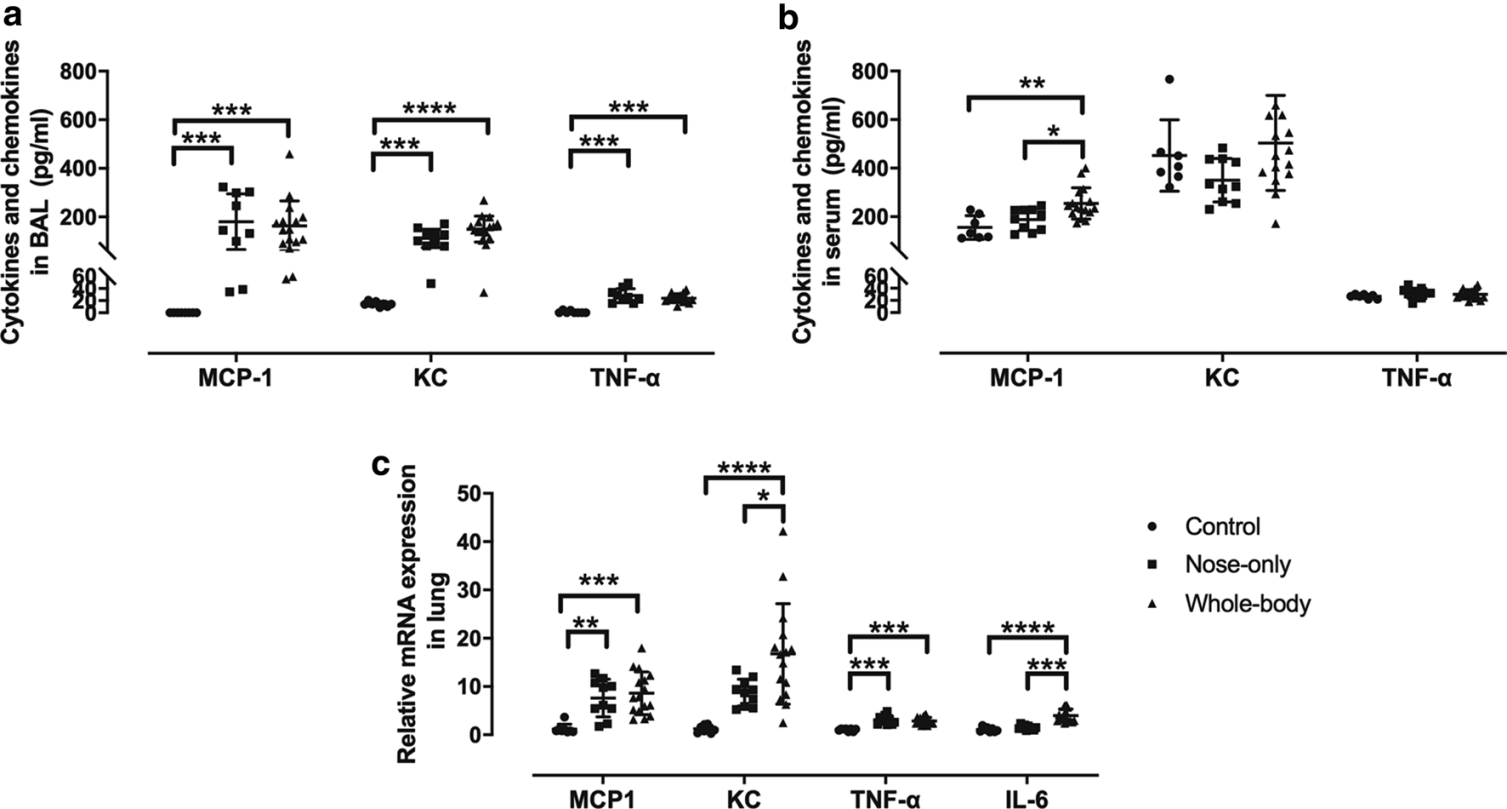
Pro-inflammatory cytokines (TNF-α and IL-6) and chemokines (MCP-1 and CXCL1) measured in **a** BAL, **b** serum and **c** lung homogenate. **a**, **b** represent protein expression in pg/ml and **c** mRNA expression determined relative to RPL27. *p < 0.05; **p < 0.01; ***p < 0.001; ****p < 0.0001. Data are expressed as median and IQR (n = 8–15)

CS exposure with both systems significantly enhanced MCP-1, CXCL1 and TNF-α protein levels in BAL to the same extent compared to control (Fig. 5a), while only serum levels of MCP-1 were significantly increased in the whole-body CS exposure system compared to others (Fig. 5b). IL-6 on protein level was under the detection limit for both BAL and serum (data not shown). The mRNA expression levels of MCP-1, CXCL1, TNF-α and IL-6 in lung homogenate were upregulated in both CS exposure systems compared to control and even more so for CXCL1 and IL-6 with whole-body compared to the nose-only CS exposure system (Fig. 5c).

### Lymphoid proliferation in mediastinal and cervical lymph nodes

**Fig. 6:**
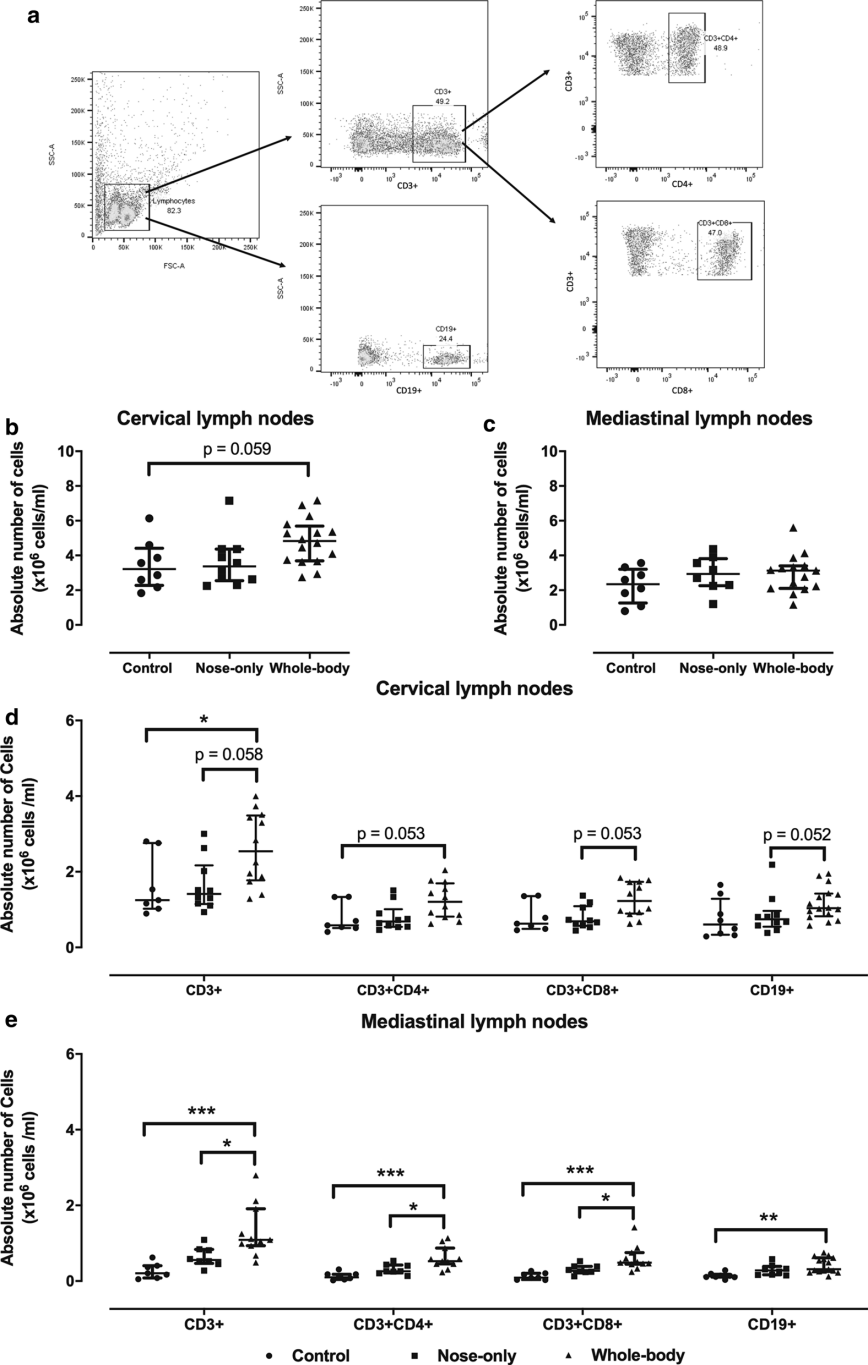
Lymphocyte proliferation in **b**, **d** cervical and **c**, **e** mediastinal lymph nodes. Flow cytometry analysis chart (A). Absolute number of cells extracted from **b** cervical and **c** mediastinal lymph nodes. Lymphocytes were characterized with flow cytometry for respective T-lymphocytes (CD3+), T-helper (CD3+ CD4+), Cytotoxic T cell (CD3+ CD8+) and B-cell (CD19+) populations in **d** cervical and **e** mediastinal lymph nodes. *p < 0.05; **p < 0.01; ***p < 0.001. Data are expressed as median and IQR (n = 8–15)

Cervical lymph nodes did not present any macroscopic differences in size between both exposure systems. However, in the whole-body exposure system a small increase in the absolute number of cells after crushing the cervical lymph nodes was noted (p = 0.059) (Fig. 6a). This was not seen in the mediastinal lymph nodes (Fig. 6b). This slight increase in total amount of cells reflected in a higher number of T lymphocytes (CD3+) and B lymphocytes (CD19+) in cervical lymph nodes with the whole-body CS exposure system in comparison with control (respectively p = 0.043 and p = 0.113) and nose-only (respectively p = 0.058 and p = 0.052) (Fig. 6c). In contrast, the percentages of CD3+ and CD19+ lymphocytes were not elevated and were more or less identical to control. The increased presence of CD3+ lymphocytes translated to more CD4+ and CD8+ lymphocytes in mice exposed to CS via the whole-body system (Fig. 6c). Unlike the cervical lymph nodes, the mediastinal lymph nodes showed a significantly higher percentage in CD3+ lymphocytes with the whole-body system compared to the nose-only CS exposure system (+ 23.7%, p = 0.034) and control (+ 27.2%, p = 0.009) (Table 2). These data were also reflected in the absolute number of lymphocytes and resulted even in a higher population of CD4+ and CD8+ T-lymphocytes despite equal percentages between groups (Fig. 6d). CD19+ lymphocytes were significantly enhanced in the whole-body CS exposure system by 7.2% (p = 0.034, Table 2) compared to control and reflected also in an increase of the absolute number of CD19+ lymphocytes (Fig. 6d).

**Table 2:** Percentage of lymphocytes in mediastinal lymph nodes

	CD3+ (%)	CD3+ CD4+ (%)	CD3+ CD8+ (%)	CD19+ (%)
*Control*	9.4 (2.4–45.8)	48.0 (44.0–49.5)	46.2 (43.4–50.9)	6.6 (3.1–9.9)
*Nose-only*	22.5 (16.0–33.3)	48.9 (45.2–50.3)	47.6 (45.8–48.9)	11.9 (5.6–14.8)
*Whole-body*	47.8 (33.5–62.2) (**) ($)	47.8 (46.2–51.0)	48.0 (42.4–49.6)	15.7 (10.1–20.1) (*)

Data are expressed as median (Q1-Q3) (n = 8–15)

Lymphocytes were characterized with flow cytometry for respective T-lymphocytes (CD3+), T-helper (CD3+ CD4+), Cytotoxic T cell (CD3+ CD8+) and B-cell (CD19+) populations

*p < 0.05; **p < 0.01 for control vs. whole-body and ^$^p < 0.05 for nose-only vs. whole-body

### Humoral immunological markers in BAL and serum

**Fig. 7:**
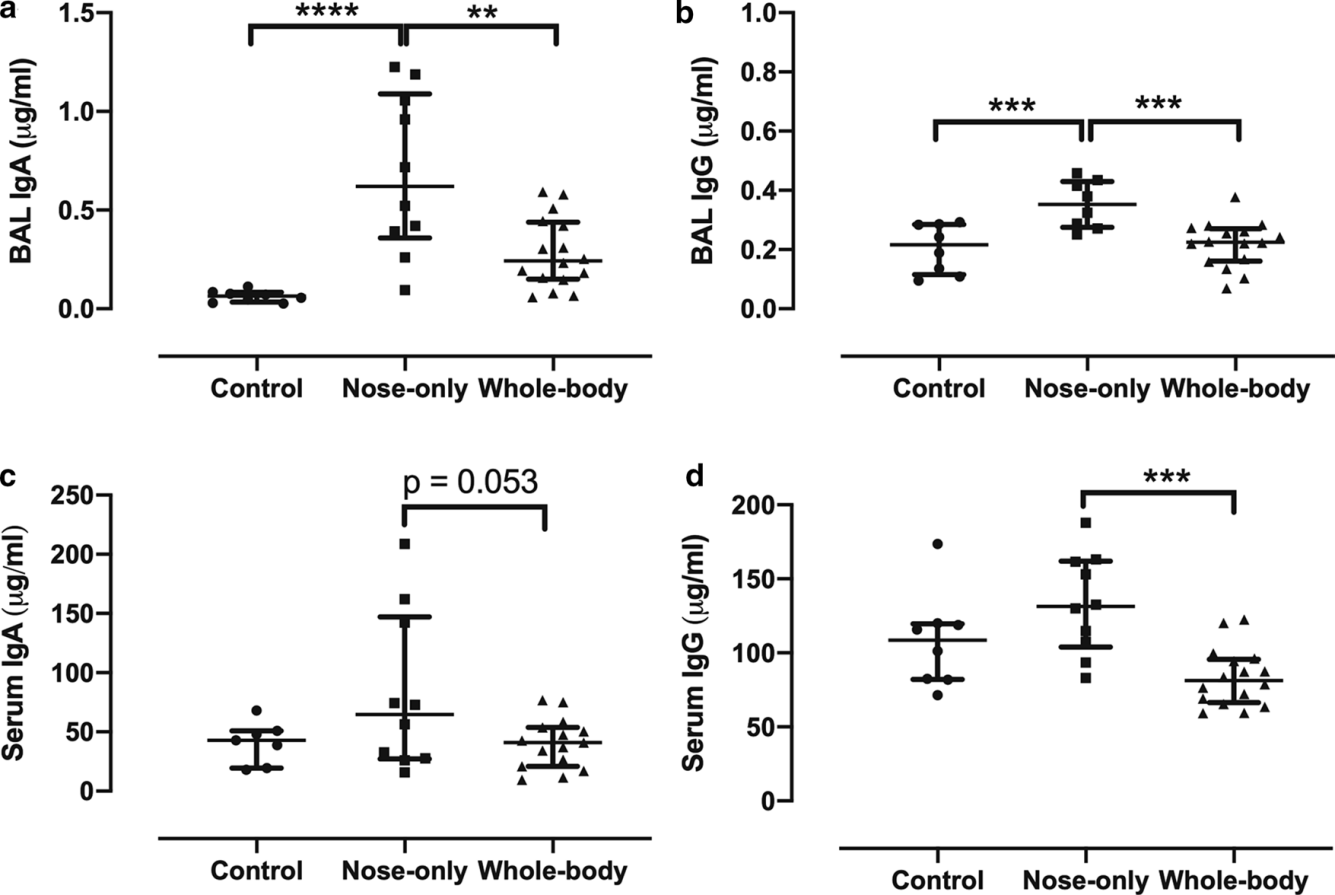
Immunoglobulins (Ig)A (**a**, **c**) and IgG (**b**, **d**) measured in BAL and serum in μg/ml. *p < 0.05; **p < 0.01; ***p < 0.001;****p < 0.0001. Data are expressed as median and IQR (n = 8–15)

IgA production in BAL was enhanced with both CS exposure systems compared to controls, especially with the nose-only system showing a significant increase in comparison to control (p < 0.0001) and whole-body CS exposure system (p = 0.0012) (Fig. 7a). Similarly, a non-significant increase in serum IgA was seen with the nose-only CS exposure system in comparison with control and whole-body CS exposure system, while the latter showed similar levels of IgA as in control (Fig. 7c). IgG production was significantly higher with the nose-only system in both BAL and serum (respectively p = 0.0015 and p = 0.0003) compared to the whole-body CS exposure system (Fig. 7b, d). The whole-body CS system showed similar IgG levels as in controls in both BAL and serum (Fig. 7b, d).

## Discussion

The standardized CS exposure protocol used in the present study, indicates that both CS exposure systems induced a similar degree of lung function impairment but more morphological alterations with the nose-only CS exposure system. Lung inflammation was more severe with the whole-body CS exposure system as shown by an enhanced lymphoid presence in BAL, lymph nodes and the upregulation of cytokine (IL-6) and chemokine (CXCL1) mRNA expression in the lung. However, this lung inflammation did not translate into enhanced immunoglobulins in the BAL or serum, which was solely observed in the nose-only CS exposure system. Importantly, this CS exposure protocol resulted in higher serum levels of cotinine in the whole-body exposure system suggesting potential uptake of CS products via other routes than just inhalation.

Up to now there have been no studies comparing whole-body with nose-only CS exposure within the same study while using a standardized exposure protocol especially at early stage of COPD. Indeed, most of the time, CS exposure models are compared with each other without having a clear knowledge regarding the effect of the CS exposure method used. Moreover, the fact that those studies have been performed separately makes it even more difficult to compare the results, notably because the CS exposure protocols differ between studies. In the present study, the CS exposure protocol was adapted so that particle matter of the CS and the carbon monoxide (CO) levels were similar in the whole-body and the nose-only systems, ensuring that mice in each exposure system were submitted to the same levels of CS. Total particulate matter was in the range of 188 ± 28.4 mg/m^3^ and CO levels were 817 ± 49 and 937 ± 50 ppm/cigarette measured in each housing compartment of respectively the nose-only and whole-body system. However, one should be aware that with the whole-body CS exposure system, it is difficult to fully control the amount of CS absorbed through the skin or gastrointestinal tract. This has been proven to induce inflammation and to alter the microflora and mucin production in the gut when using the whole-body exposure system [14, 15]. Actually, the twofold higher cotinine serum levels we found in the whole-body system supports the uptake of CS products through absorption via a different route than the lungs. It is clear that in the whole-body exposure system, mice are exposed to CS not only through inhalation but also via their skin and grooming. This might have contributed to the elevated cotinine serum levels in whole body exposure system. Conversely, the nose-only CS exposure system allows a better controllability on the amount of CS inhaled and circumvents the influence of its absorption through the skin. Finally, one aspect that should not be neglected in these models relates to the breathing pattern following CS as this might impact CS internal load. While data in literature regarding breathing frequency, tidal volume and minute ventilation with CS exposure are inconsistent and mainly refer to rodent data other than mice [16–18], we cannot guarantee that the breathing pattern of the mice in the whole-body and the nose-only exposure systems was similar. There is still a possibility that alterations in the breathing pattern of the animals in the whole-body exposure system might have favored a higher uptake of CS products and contributed for a part to the higher serum levels of cotinine.

Currently, only one study has performed a comparison between the nose-only and whole-body system, but with some major differences compared to our study [19]. Shu et al*.* used a mouse model combining CS exposure with lipopolysaccharides (LPS), where mice were exposed two times to an LPS-instillation at day 0 and 14 followed with an intense CS exposure protocol using 18 cigarettes twice a day for 10 weeks (6 days/week). In our model, we found after 14 weeks of CS exposure alone (6 cigarettes twice/day) a 25% less increase in FRC and no enhanced chord compliance compared to the data reported by Shu et al. However, in agreement with Shu et al*.*’s data, no differences in lung function were observed between the two CS exposure systems. Although only discrete alterations were found in the lung histology in our study compared to the severe changes such as bronchial wall thickening, emphysema, goblet cell hyperplasia and even pulmonary bullae formation reported in Shu et al*.*’s study [19], both studies indicated no differences between the two CS exposure systems. It is worth to mention, that this type of severe alterations in lung histology as shown in Shu et al*.*’s study usually occur after 24 weeks of CS exposure [20–22]. The intense CS exposure protocol combined with LPS instillations might explain the presence of these alterations at an earlier time point. Finally, our study indicated that the mean airspace enlargement was slightly enhanced in the nose-only exposure system compared to whole-body, but this enlargement was not significantly different compared to control. Taken together, these data suggest that nose-only and whole-body CS exposure systems lead to the same degree of lung function impairment and histological alterations.

Interestingly, we also looked at desmosine, a crosslinker between elastin fibers that is usually used as a biomarker for elastin breakdown, and that has been found to be enhanced in the BAL fluid at this stage of emphysema [23, 24]. Desmosine in BAL has also been reported to correlate with mean airspace size and with BAL neutrophils [25, 26]. Up to now, no studies have compared (iso-)desmosine levels in nose-only and whole-body CS exposure systems. In our experiment, while desmosine was undetectable in the BAL fluid with both CS exposure systems, a significant increase in iso-desmosine and total desmosine in serum was observed with the whole-body CS exposure system compared to the nose-only CS exposure system and to control. However, since the MMP12/TIMP1 mRNA expression ratio in the lungs were similar between both CS exposure systems, this suggests that lung proteolysis was similar and that the enhanced serum desmosine, in the whole-body CS exposure system, is likely to come from another source i.e. elastin degradation of the skin [27].

Regarding inflammation, in agreement with Shu et al*.* [19], no differences in BAL macrophages or neutrophils were noticeable between the two CS exposure systems. By contrast, the increase in BAL lymphocytes was significantly more pronounced with the whole-body CS exposure system compared to nose-only exposure and controls. This enhanced lymphocyte presence with the whole-body CS exposure system might suggest a progressive implication of the adaptive immunity with further worsening of lung remodeling and inflammation. mRNA expression of CXCL1 and IL-6 in the lung was already more increased in the whole-body CS exposure system in comparison with nose-only, but BAL and serum protein levels of pro-inflammatory cytokines, particular CXCL1 and IL-6, were similarly enhanced in both CS exposure systems, as shown also by Shu et al. [19]. Overall, these data indicate an enhanced lymphocyte inflammation in the lungs with the whole-body CS exposure system.

Furthermore, both CS exposure systems demonstrated an equal amount of lymphoid inflammation and aggregates. In an attempt to categorize the lung inflammation of each CS exposure system, cytokines specific for Th1 (IFN-γ, IL-12p40), Th2 (IL-4, IL-13), Th17 (IL-17, IL-23) and regulatory T lymphocytes (Treg; IL-10, TGF-β) response were measured via mRNA expression in lung homogenate. Actually, most animal models of emphysema with CS exposure reported a type 1 mediated inflammation with Th1/Th17 immunity [28, 29], while other studies that use CS extracts [30] for in vivo or in vitro experiments showed a polarization towards a type 2 mediated inflammation with Th2 immunity (reviewed in [31]). In fact, CS can suppress the expression of IL-12 and IL-23 in dendritic cells, needed for a Th1/Th17 differentiation [32]. In addition, CS can also be used as an adjuvant to provoke an enhanced Th2 immune response when combined with allergens [30, 33]. In our study, we showed a similar enhanced expression of IL-4, IL-13, IL-10 and TGF-β in the lungs with no changes in IFN-γ and IL-17 with both CS exposure systems. This corresponds to a type 2 mediated inflammation with a Th2 and Treg immune response which is surprising in the presence of IL-12 and IL-23. It stands to fact that this balance can later shift towards a full Th1/Th17 response with production of IFN-γ and IL-17, when CS exposure will induce more damage to the lungs [28, 29, 34] and might even persist after smoking cessation [35–37]. In human COPD, lymphocyte inflammation is mainly driven by a Th1 immune response with an enhanced IFN-γ production in both helper (CD4+) and cytotoxic (CD8+) T lymphocytes, that increased with COPD severity [38, 39]. The reason why this was not observed with any of our CS exposure systems might be related to the duration and intensity (number of cigarettes, filtered versus unfiltered cigarettes) of the CS exposure protocol. Nevertheless, both IFN-γ and IL-13 inflammation can induce emphysema [40, 41]. In summary, in our study both CS exposure systems induce a Th2/Treg induced inflammation in the lungs, likely related to the duration and intensity of our CS exposure protocol.

Our data shows an enhanced presence of (CD3+) T- and (CD19+) B-lymphocytes in the cervical and mediastinal lymph nodes with the whole-body CS exposure system. This suggests a role for sensitization at the skin or gastrointestinal tract. Indeed, Pollaris et al*.* demonstrated for toluene diisocyanate that dermal sensitization, before repeated intranasal challenges, could induce an aggravated lymphocyte proliferation in lymph nodes and even airway hyperreactivity [42]. Thus, in our study, sensitization to CS with the whole-body system might have provoked an aggravated inflammatory response in the lungs during CS exposure. However, for the time being, there are no data proving if this assumption is valid. On the other hand, opposite immunological effects of CS exposure have been reported between the CS exposure systems. Robbins et al. demonstrated, in a nose-only CS exposure system, a decrease in number of dendritic cells (DC) in the lungs and an inhibition of their maturation, which suppresses the proliferation of lymphocytes in the lymph nodes [43]. However, Demoor et al. showed, in a whole-body CS exposure system, an increase in lung DCs already at 4 weeks followed by an enhanced lymphocyte proliferation in the lymph nodes which was almost 3 times higher than normal after 24 weeks of CS exposure [44]. In human smokers, DCs are also highly recruited to the lungs but the exposure to CS makes them almost ‘non-functional’ and inhibit their antigen-presenting capacity [45]. These data let us postulate that CS exposure with the whole-body system would induce an immunological response presumably involving more lymph nodes and systemic activation. However, it remains difficult to fully support this postulate with the current data as the smoking protocols were different and it seems not fair to compare data obtained with 4 against 20 unfiltered cigarettes/day [43, 44].

Humoral immunological markers (e.g. IgA and IgG) were enhanced in BAL and serum of mice exposed to the nose-only CS exposure system in comparison with the whole-body system and control. These data are, however, inconsistent with the increased B-lymphocytes in the lymph nodes of mice exposed to the whole-body CS exposure system and could be related to the maturation or migration of B-lymphocytes in the two systems as we only measured the general CD19+ B-lymphocyte marker and not the markers for plasma B lymphocytes. However, a significant increase in IgA, IgM and IgG in BAL, but not in serum was previously reported when using the whole-body exposure system with CS from cigarettes without filter [46]. In our study, this increase in Ig’s might be related to the fact that CS in the nose-only system is likely denser inhaled than in the whole-body system.

## Conclusion

The current standardized CS exposure protocol resulted in a higher internal load of serum cotinine in the whole-body exposure system and was associated with an increased lymphocytic lung inflammation and proliferation in adjacent lymph nodes. Both exposure systems resulted in a similar lung function impairment, but clear differences could be highlighted between the two models in terms of lung inflammation, remodelling and potential sensitization to CS. Researchers should be aware of these differences when designing their future studies for an early intervention in COPD.

## Data Availability

All data generated or analyzed during this study are included in this published article and its supplementary information files. Datasets used or analyzed during the current study are available from the corresponding author on reasonable request.

## Abbreviations

COPD: Chronic obstructive pulmonary disease
CS: Cigarette smoke
CO: Carbon monoxide
FEV_1_: Forced expiratory volume in 1 s
FVC: Forced vital capacity
Th: T helper
TLF: Tertiary lymphoid follicles
FRC: Functional residual capacity
IC: Inspiratory capacity
TLC: Total lung capacity
PV-loop: Pressure–volume maneuver
C chord: Chord compliance between 0 and 10 cmH_2_O
FV-loop: Fast-volume maneuver
BAL: Bronchoalveolar lavage
Lm: Mean linear intercept
LC–MS/MS: Liquid chromatography–mass tandem–mass spectrometry
Ig: Immunoglobulin
RPL27: Ribosomal protein L27
MMP12: Matrix metalloproteinase 12
TIMP1: Tissue inhibitor of MMP 1
CXCL1: C-X-C motif ligand 1
TNF: Tumor necrosis factor
IFN: Interferon
IL: Interleukin
TGF: Tumor growth factor
AUC: Area under the curve
LPS: Lipopolysaccharides
DC: Dendritic cell
